# Association of anesthesia strategies with outcomes in endovascular treatment for distal and medium vessel occlusions: A propensity score-matched analysis of the MR CLEAN registry and meta-analysis

**DOI:** 10.1093/esj/23969873251352406

**Published:** 2026-01-01

**Authors:** Mohamed F Doheim, Robrecht R M M Knapen, Diederik W J Dippel, Julie Staals, Jeannette Hofmeijer, Adriaan C G M van Es, Jonathan M Coutinho, Christiaan van der Leij, Raul G Nogueira, Robert J van Oostenbrugge, Wim H van Zwam

**Affiliations:** Department of Neurology, Stroke Institute, University of Pittsburgh Medical Center, Pittsburgh, PA, USA; Department of Neurology, Maastricht University Medical Center +, Maastricht, The Netherlands; School for Cardiovascular Diseases Maastricht (CARIM), Maastricht University, The Netherlands; Department of Neurology, Maastricht University Medical Center +, Maastricht, The Netherlands; School for Cardiovascular Diseases Maastricht (CARIM), Maastricht University, The Netherlands; Department of Radiology and Nuclear Medicine, Maastricht University Medical Center +, Maastricht, The Netherlands; Department of Neurology, Erasmus University Medical Center, Rotterdam, The Netherlands; Department of Neurology, Maastricht University Medical Center +, Maastricht, The Netherlands; Department of Neurology, Rijnstate Hospital, Arnhem, The Netherlands; Department of Clinical Neurophysiology, University of Twente, Enschede, The Netherlands; Department of Radiology and Nuclear Medicine, Leiden University Medical Center, The Netherlands; Department of Neurology, Amsterdam UMC, University of Amsterdam, Amsterdam, The Netherlands; Department of Radiology and Nuclear Medicine, Maastricht University Medical Center +, Maastricht, The Netherlands; GROW, School for Oncology and Reproduction, Maastricht University, Maastricht, The Netherlands; Department of Neurology, Stroke Institute, University of Pittsburgh Medical Center, Pittsburgh, PA, USA; Department of Neurology, Maastricht University Medical Center +, Maastricht, The Netherlands; School for Cardiovascular Diseases Maastricht (CARIM), Maastricht University, The Netherlands; School for Cardiovascular Diseases Maastricht (CARIM), Maastricht University, The Netherlands; Department of Radiology and Nuclear Medicine, Maastricht University Medical Center +, Maastricht, The Netherlands

**Keywords:** Anesthesia, stroke, endovascular treatment

## Abstract

**Background:**

Recent trials did not demonstrate the benefit of endovascular therapy (EVT) for distal or medium vessel occlusions (DMVOs), raising questions about factors influencing outcomes. Anesthesia choice may play a role, yet its impact remains unclear. This study assessed general anesthesia (GA) versus non-GA in EVT for DMVOs, evaluating procedural, functional, and safety outcomes.

**Patients and methods:**

Patients undergoing EVT for AIS due to anterior DMVOs in the middle cerebral artery (MCA-M2, M3, M4) and anterior cerebral artery (ACA-A1, A2, A3) from the MR CLEAN registry between March 2014 and December 2018 were included. They were stratified into GA and non-GA groups, with propensity score matching employed to adjust for differences in baseline risk. Primary outcomes included functional outcomes at 90 days, assessed by ordinal regression analysis of modified Rankin Scale (mRS) scores at 90 days, and recanalization rates measured by Thrombolysis in Cerebral Infarction (TICI) scores. Secondary outcomes included dichotomized mRS scores, death at 90 days, and symptomatic intracranial hemorrhage (sICH). A systematic review and meta-analysis of relevant DMVO studies with a random effects model was performed. This study was registered with PROSPERO (CRD42024607294).

**Results:**

Among 5193 patients in the registry, 657 were eligible for our study, with 506 in the non-GA group, and 151 in the GA group. The median age was 73 years (IQR 64–81) in the non-GA group and 73 years (IQR 61–80) in the GA group (*p* = 0.35). The proportion of male patients was 50.2% in the non-GA group and 57.0% in the GA group (*p* = 0.15). In the matched cohort (*n* = 170), recanalization rates were higher in the GA group compared to the non-GA group (excellent recanalization rates (TICI2c/3): 61.0% vs 32.1%; OR 3.31, 95% CI (1.74–6.29), *p* < 0.001). There were no significant differences in the overall distribution of functional outcomes at 90 days (common OR 0.93, 95% CI (0.54–1.56), *p* = 0.77). Mortality was comparable between groups (34.1% vs 31.8%; OR 1.11, 95% CI (0.59–2.11), *p* = 0.74), and there was no significant difference in sICH (12.9% vs 5.9%; OR 0.42, 95% CI (0.14–1.27), *p* = 0.12). The systematic review and meta-analysis included six studies with a total of 3521 patients. The pooled analysis indicated that GA was associated with significantly lower rates of excellent functional outcomes (mRS 0–1: OR 0.74, 95% CI (0.58–0.94), *p* = 0.01) and higher mortality (OR 1.36, 95% CI (1.07–1.74), *p* = 0.01) compared to the non-GA at 90 days.

**Discussion and conclusion:**

In the MR CLEAN Registry, GA was associated with higher recanalization rates during EVT, but this technical advantage did not translate into improved 90-day functional outcomes. Our meta-analysis further indicated that non-GA strategies were associated with better functional recovery and lower mortality. These associations, however, warrant cautious interpretation given potential unmeasured confounders, including blood pressure management and conversion from non-GA to GA. Broad categorization of anesthesia as GA versus non-GA overlooks critical factors such as agent selection, physiological targets, and intraoperative monitoring, which may substantially impact cerebral perfusion and outcomes. Further prospective randomized studies with detailed anesthetic data and expert input are needed to refine these findings and guide clinical practice.

## Graphical Abstract

**Graphical abstract:**
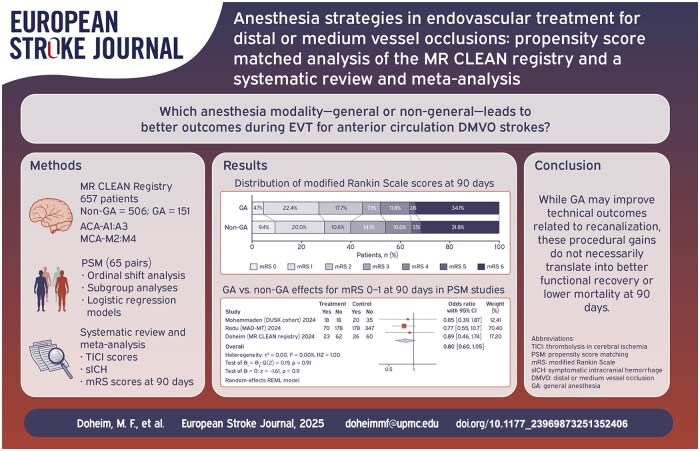

## Introduction

Acute ischemic stroke (AIS) remains a leading cause of disability and mortality worldwide, with endovascular treatment (EVT) emerging as the standard of care for eligible AIS patients.^1–4^ However, several factors, including the use of general anesthesia (GA) during EVT, may influence procedural success and clinical outcomes. Despite extensive research, the optimal anesthesia strategy for EVT remains debated.^5–10^ GA has been associated with immobility and better imaging quality, potentially improving technical outcomes such as recanalization rates. Conversely, concerns exist about potential peri-procedural hypotension and treatment delays caused by GA, negatively affecting functional recovery.^10–14^ As a result, non-GA strategies, such as conscious sedation and local anesthesia, have gained traction as potentially more favorable for functional outcomes. Studies examining these approaches have reported conflicting results, with some suggesting a procedural benefit for GA and others indicating better functional outcomes with non-GA techniques.^6,8,15–17^

Distal or medium vessel occlusions (DMVOs), including occlusions in the middle cerebral artery (MCA-M2, M3, M4), anterior cerebral artery (ACA-A1, A2, A3), and posterior cerebral artery (P1, P2, P3), present unique challenges and opportunities in the context of EVT.^18,19^ These occlusions are often more technically demanding due to their smaller vessel size, tortuosity, and more distal location, complicating navigation and clot retrieval.^18,19^

Recent trials, including DISTAL and ESCAPE-MeVO, have shown neutral results, and did not demonstrate a significant benefit of EVT for DMVOs. These findings have sparked considerable debate regarding the factors influencing EVT outcomes. Among these, anesthesia choice has emerged as a critical consideration. For instance, ESCAPE-MeVO reported higher rates of serious adverse events, such as pneumonia and infections, possibly influenced by procedural sedation. GA use varied widely, from 9.1% in ESCAPE for proximal large vessel occlusion 41.3% in ESCAPE-MeVO and 49.6% in DISTAL, reflecting a growing preference for GA in DMVOs, though its safety, and efficacy remain uncertain.^20–22^ This study aimed to address these gaps by comparing GA and non-GA in EVT for anterior circulation DMVOs (MCA-M2, M3; ACA-A1, A2) within the MR CLEAN registry and conducting a systematic review and meta-analysis of six studies (3521 patients) involving anterior and posterior DMVOs (MCA-M2, M3, M4; ACA-A1, A2, A3; PCA-P1, P2, P3) to comprehensively evaluate GA versus non-GA strategies.

## Methods

### Study design and population

The primary cohort study was reported in accordance with the STROBE (Strengthening the Reporting of Observational Studies in Epidemiology) statement. In addition, we conducted a systematic review and meta-analysis following a predefined protocol, in line with the Preferred Reporting Items for Systematic Reviews and Meta-Analyses (PRISMA) guidelines and the Cochrane Handbook for Systematic Reviews.^23,24^ This study conducted an analysis of data from the MR CLEAN (Multicenter Randomized Clinical trial of Endovascular treatment for Acute ischemic stroke in the Netherlands) registry between March 2014 and December 2018, examining the impact of anesthesia type (GA vs non-GA) on outcomes in patients undergoing EVT for AIS. This study specifically focused on anterior circulation DMVOs involving the MCA-M2, M3 and ACA-A1, A2, and excluded posterior DMVOs (PCA) to avoid potential selection bias among other reported limitations.^25,26^

Patients were stratified into two groups based on the type of anesthesia administered during EVT: GA or non-GA. GA was defined as a state of unconsciousness requiring advanced airway protection, including tracheal intubation or laryngeal mask placement, along with mechanical ventilation and sedation using agents such as propofol or midazolam. Non-GA encompassed two approaches: conscious sedation (CS) and local anesthesia (LA). CS was defined as the administration of systemic sedative medications with the intention of keeping the patient comfortable while maintaining spontaneous breathing, without requiring advanced airway support. LA was defined as the use of a local anesthetic at the puncture site, without the administration of systemic sedatives. Anesthetic management strategies varied by center, with institutional preferences guiding the choice between GA, CS, or LA. However, one interventionist in a center where LA was the standard approach routinely preferred CS. The specific protocols for GA and CS, including sedative choice, ventilation settings, and hemodynamic management, were determined by the treating physicians based on individual patient needs and institutional practice.

### Data variables

The data collected for this study included age, gender, National Institutes of Health Stroke Scale (NIHSS) at baseline, and pre- modified Rankin Scale (mRS) scores. Intravenous Thrombolysis (IVT) administration was recorded. Medical history variables included previous stroke, atrial fibrillation, hypertension, hypercholesterolemia, diabetes mellitus, and current smoking. Anticoagulation and antiplatelet use were also documented. The site of occlusion was identified as MCA-M2, MCA-M3, ACA-A1, or ACA-A2. Collateral grading and Alberta Stroke Program Early CT Score (ASPECTS) scores were noted, along with information on whether patients were transferred from a primary stroke center.

Procedural outcomes included stroke onset-to-groin time, procedure time, first pass effect (FPE); defined as the achievement of complete or near-complete recanalization (Thrombolysis in Cerebral Infarction (TICI) 2c/3) with a single pass, modified FPE (mFPE); defined as the achievement of complete or near-complete recanalization (TICI 2b-3) with a single pass, and recanalization outcomes; based on TICI grades. Successful recanalization was defined as TICI 2b-3, excellent recanalization as TICI 2c/3, and complete recanalization as TICI 3. All imaging was evaluated by an independent core laboratory. Vessel dissection, perforation, and embolization in new territories were recorded. Clinical outcomes included NIHSS at 24–48 h and mRS scores: excellent outcomes (mRS 0–1), functional independence/good functional outcome (mRS 0–2), and fair functional outcomes (mRS 0–3). Safety outcomes included rates of mortality at 90 days, symptomatic intracranial hemorrhage (sICH), and pneumonia.

### Statistical analysis for the MR CLEAN cohort

#### Overall unmatched analysis

Baseline characteristics between the GA and non-GA groups were compared using chi-square tests for categorical variables and Mann-Whitney *U* tests for continuous variables. To further refine the analysis, logistic regression models were used to assess the association between the type of anesthesia (GA vs non-GA) and both primary and secondary outcomes. Adjustments were made for variables including age, baseline NIHSS, ASPECTS, site of occlusion, and pre-mRS. An adjusted ordinal shift analysis of mRS scores at 90 days was conducted to evaluate the overall distribution of outcomes across the entire mRS range (0–6). The results of this analysis were expressed as adjusted common odds ratios (acOR), reflecting the likelihood of an ordinal shift toward better functional outcomes.

#### Propensity score matching (PSM)

Propensity score matching (PSM) was performed via the psmatch2 in Stata to address baseline imbalances and minimize confounding. Nearest neighbor matching with a 1:1 ratio, without replacement, and a caliper width of 0.2 standard deviations of the logit of the propensity score was employed. The matching criteria included age, baseline NIHSS score, ASPECTS, site of occlusion, and pre-mRS, ensuring that both groups were comparable in terms of these covariates and reducing confounding.

#### Subgroup analysis

In addition to the ordinal shift analysis, subgroup interaction analyses in the matched groups were performed for various clinically relevant subgroups, including sex, age (<80 vs ⩾80), IVT administration, ASPECTS (⩽8 vs >8), and transfer status (mothership vs transfer). Moreover, the impact of NIHSS severity was evaluated by comparing NIHSS >10 versus NIHSS ⩽10 subgroups. The results from both the ordinal shift and subgroup analyses were reported as adjusted OR (aOR) with corresponding 95% confidence intervals (CIs). Statistical significance was set at *p* < 0.05 for all analyses. All analyses were conducted using Stata software (StataCorp LLC, College Station, TX) and R software (R Foundation for Statistical Computing, Vienna, Austria).

### Systematic review

This study was registered with PROSPERO (CRD42024607294). A comprehensive literature search was conducted across multiple databases, including PubMed, Embase, and the Cochrane Library, covering the period from January 2010 to October 2024. The search strategy employed a combination of keywords such as “distal medium vessel occlusion,” “endovascular treatment,” and “acute ischemic stroke.” Additionally, reference lists of relevant articles were meticulously reviewed to identify any additional studies that met the inclusion criteria. Studies were selected based on predefined inclusion criteria. Eligible studies were those that reported on patients with DMVO involving the MCA-M2, M3, M4, ACA-A1, A2, A3) or PCA-P1, P2, P3 who underwent EVT. Furthermore, studies needed to provide sufficient data to calculate ORs for the outcomes of interest and did not restrict their criteria to occlusions in M2. The selection process was carried out independently by two reviewers, and any discrepancies were resolved through discussion and consensus. Data extraction was performed independently by two reviewers who collected information on study characteristics, patient demographics, and clinical outcomes.

### Meta-analysis

#### Overall pooled analysis

For the meta-analysis, effect sizes for each study were calculated as logit-transformed ORs using random effects and Mantel-Haenszel weighting. The between-study variance in the random-effects model was estimated via restricted maximum likelihood, with 95% CIs provided for precision. To assess heterogeneity among the included studies, Higgin’s *I*^2^ statistic was employed, indicating the proportion of total variability in effect estimates due to heterogeneity rather than sampling error.^27^ The τ^2^ values in the forest plots represented the true variance in effect sizes, also estimated through restricted maximum likelihood. The primary outcomes of interest included mRS scores of 0–1 and 0–2, mortality at 90 days, sICH, successful reperfusion (TICI 2b-3), and FPE. For Mehta et al.,^28^ good functional outcomes were defined as mRS 0–3 instead of mRS 0–2, while sICH was assessed using the reported ICH rates as a surrogate. The event numbers used for the pooled analysis were extracted from *N* (%) for both functional outcomes and sICH events.

#### Sensitivity analysis for meta-analysis

In addition to the presented analysis from this study that utilized PSM, Mohammaden et al.^29^ and Radu et al.,^16^ included in the meta-analysis, also employed this method, while the remaining three reported overall analyses without PSM.^17,28,30^ In our meta-analysis, studies were classified into two categories: those utilizing PSM and those providing overall analyses without it. This classification allowed for subgroup analyses based on PSM and overall effect estimates, enhancing our understanding of the impact of PSM at the study level. Additionally, leave-one-out sensitivity analyses were performed to evaluate the robustness of the overall findings. This method involved systematically removing one study at a time from the meta-analysis to assess how each study influences the overall results. This approach enhanced the credibility of the findings by demonstrating that the overall results were not unduly dependent on any single study.^27,31^

#### Publication bias and meta-regression

Assessment of publication bias and meta-regression was not performed due to the inclusion of fewer than 10 studies, as these analyses require a larger sample size for reliable and valid results.^31,32^ All analyses were conducted using Stata software (StataCorp LLC, College Station, TX).

## Results

### Baseline characteristics

#### Unmatched overall cohort

Among 5193 patients in MR CLEAN registry, 657 patients met the eligibility criteria, with 506 in the non-GA group, and 151 in the GA group. Baseline stroke severity, as assessed by the NIHSS, was higher in the GA group, with a median score of 13 (IQR 9–17) compared to 10 (IQR 6–16) in the non-GA group (*p* = 0.003; Table 1). Additionally, the pre-stroke-mRS scores were different between the groups, with a higher percentage of patients having a pre-mRS score of 0 in the non-GA group (62.5% vs 52.5%; *p* = 0.02). The ASPECTS scores were also slightly lower in the GA group, with a median score of 9 (IQR 8–10) compared to 10 (IQR 9–10) in the non-GA group (*p* < 0.001). Other baseline characteristics showed no significant differences between the groups. The median age was 73 years (IQR 64–81) in the non-GA group and 73 years (IQR 61–80) in the GA group (*p* = 0.35). The proportion of male patients was 50.2% in the non-GA group and 57.0% in the GA group (*p* = 0.15). The rates of hypertension, diabetes mellitus, and atrial fibrillation were similar between the groups. The site of occlusion was predominantly MCA-M2 in both groups, with 98.0% in the non-GA group and 94.7% in the GA group (*p* = 0.03). The rates of IVT administration were also comparable, with 66.1% in the non-GA group and 67.6% in the GA group (*p* = 0.74).

**Table 1.** Baseline patient characteristics and outcomes in overall and matched cohort.

Characteristics and outcomes	Overall			Matched		
	Non-GA (*N* = 506)	GA (*N* = 151)	*p*-value	Non-GA (*N* = 85)	GA (*N* = 85)	*p*-value
Age—median (IQR)*	73 (64–81)	73 (61–80)	0.35	72 (63–81)	74 (66–80)	0.90
Male	254 (50.2)	86 (57.0)	0.15	41 (48.2)	48 (56.5)	0.28
NIHSS baseline—median (IQR)*	*N* = 504 10 (6.0–16.0)	*N* = 143 13 (9.0–17.0)	0.003	10 (7–16)	12 (8–16)	0.58
Pre-mRS—*n* (%)*	*N* = 496	*N* = 143	0.02	*N* = 85	*N* = 85	0.89
0	310 (62.5)	75 (52.5)		47 (55.3)	47 (55.3)	
1	87 (17.5)	22 (15.4)		12 (14.1)	15 (17.7)	
2	43 (8.7)	17 (11.9)		8 (9.4)	6 (7.1)	
>2	56 (11.3)	29 (20.3)		18 (21.2)	17 (20.0)	
IVT given—*n* (%)	333 (66.1)	102 (67.6)	0.74	62/84 (73.8)	53/85 (62.4)	0.11
Medical history—*n* (%)						
Previous stroke	100/499 (20.0)	32/151 (21.2)	0.76	18/82 (22)	19/85 (22.4)	0.95
Atrial fibrillation	132/497 (26.6)	40/150 (26.7)	0.98	23/83 (27.7)	26/85 (30.6)	0.68
Hypertension	281/492 (57.1)	84/150 (55)	0.81	41/80 (51.3)	50/84 (59.5)	0.29
Hypercholesteremia	154/487 (31.6)	57 (38.3)	0.13	24/80 (30.0)	35/85 (41.2)	0.13
Diabetes mellitus	89/503 (17.7)	28 (18.5)	0.81	14/83 (16.9)	17/85 (20.0)	0.60
Current smoking	91/400(22.8)	27/106 (25.5)	0.56	12/69 (17.4)	15/64 (23.4)	0.39
Anticoagulation use—*n* (%)	35/503(7.0)	11/149 (7.4)	0.86	5/85 (5.9)	9/85 (10.6)	0.26
Antiplatelet use—*n* (%)	163/502 (32.5)	50/149 (33.6)	0.80	24 (28.8)	28 (33.3)	0.50
Site of occlusion—*n* (%)*	506	151	0.03	85	85	0.17
MCA-M2	496 (98.0)	143 (94.7)		82 (96.5)	81 (95.3)	
MCA-M3	3 (0.6)	5 (3.3)		0 (0.0)	3 (3.5)	
ACA-A1	2 (0.4)	2 (1.3)		1 (1.2)	0 (0.0)	
ACA-A2	5 (0.99)	1 (0.66)		2 (2.4)	0 (0.0)	
Collaterals—*n* (%)	493	149	0.90	81	83	0.71
0	14 (2.8)	3 (2.0)		3 (3.7)	1 (1.2)	
1	150 (30.4)	49 (32.9)		28 (34.6)	27 (32.5)	
2	206 (41.8)	61 (40.9)		29 (35.8)	34 (41.0)	
3	123 (25)	36 (24.2)		21 (25.9)	21 (25.3)	
ASPECTS—median (IQR)*	*N* = 500 10 (9–10)	*N* = 150 9 (8.0–10)	<0.001	10 (9–10)	9 (8–10)	0.42
Transfer from primary stroke center—*n* (%)	199/506 (39.3)	69/151 (45.7)	0.16	39/85 (45.9)	37/85 (43.5)	0.76
Procedural outcomes						
Time to groin—median (IQR), min	*N* = 500 189 (131–253)	*N* = 149 204 (157–254)	0.07	*N* = 84 183 (146–256)	*N* = 84 195 (157–245)	0.78
Procedure time—median (IQR), min	*N* = 480 51 (35–70)	*N* = 142 50 (30–82)	0.98	*N* = 79 55 (36–70)	*N* = 81 52 (31–82)	0.80
FPE—*n* (%)	134/482 (27.8)	41/145 (28.3)	0.91	16/81 (19.8)	24/82 (29.3)	0.16
mFPE—*n* (%)	183/482 (38.0)	54/145 (37.2)	0.87	24/81 (29.6)	28/82 (34.2)	0.56
Successful recanalization—*n* (%)	312/482 (64.7)	103/145 (71.0)	0.16	41/81 (50.6)	60/82 (73.2)	0.003
Excellent recanalization—*n* (%)	211/482 (43.8)	79/145 (54.5)	0.02	26/81 (32.1)	50/82 (61.0)	<0.001
Complete recanalization—*n* (%)	163/482 (33.8)	66/145 (45.5)	0.01	22/81 (27.2)	43/82 (52.4)	0.001
Vessel dissection—*n* (%)	6/465 (1.3)	2/137 (1.5)	>0.999	2/80 (2.5)	1/79 (1.3)	0.57
Perforation—*n* (%)	8/466 (1.7)	2/137(1.5)	>0.999	4/81 (4.9)	0/79 (0.0)	0.12
Embolization in new territories—*n* (%)	13/471 (2.8)	5/138 (3.6)	0.60	1/82 (1.2)	3/80 (3.8)	0.30
Functional outcomes	mRS *N* = 460	mRS *N* = 136		mRS *N* = 85	mRS *N* = 85	
NIHSS at 24–48 h—median (IQR)	*N* = 492 7.0 (2.0–15)	*N* = 136 9.0 (5.0–16)	0.02	*N* = 85 8 (3–15)	*N* = 79 8 (4–16)	0.43
mRS 0–1—*n* (%)	157 (34.1)	27 (19.9)	0.002	25 (29.4)	23 (27.1)	0.73
mRS 0–2—*n* (%)	225 (48.9)	55 (40.4)	0.08	34 (40.0)	38 (44.7)	0.54
mRS 0–3—*n* (%)	291 (63.3)	68 (50.0)	0.01	46 (54.1)	44 (51.8)	0.76
Safety outcomes						
Mortality at 90 days—*n* (%)	190 (23.7)	48 (35.3)	0.01	27 (31.8)	29 (34.1)	0.74
sICH—*n* (%)	36/506(7.1)	9/151 (6.0)	0.62	11 (12.9)	5 (5.9)	0.12
Pneumonia—*n* (%)	53/506 (10.5)	16/151 (10.6)	0.99	18/85 (21.2)	13/85 (15.3)	0.32

^*^Used for propensity score matching.

#### Matched cohort

After matching 85 patients from each group, there were no significant differences in baseline characteristics (Table 1). The median NIHSS score at baseline was 10 (IQR 7–16) in the non-GA group and 12 (IQR 8–16) in the GA group (*p* = 0.58). The pre-mRS scores were evenly distributed, with no significant differences between the groups (*p* = 0.89). The rates of IVT administration were 73.8% in the non-GA group and 62.4% in the GA group (*p* = 0.11). The site of occlusion was predominantly MCA-M2 in both groups, with 96.5% in the non-GA group and 95.3% in the GA group (*p* = 0.17). The ASPECTS scores were similar, with a median score of 10 (IQR 9–10) in the non-GA group and 9 (IQR 8–10) in the GA group (*p* = 0.42).

#### Meta-analysis

The meta-analysis included six studies with a total of 3521 patients.^16,17,28,29^ The studies varied in design, period, and eligibility criteria (Supplemental Table S1). Mohammaden et al. (DUSK Cohort)^29^ included 366 patients with DMVO involving the MCA-M3/4, ACA-A2/3, or PCA-P1/P2-3 who underwent EVT, with PSM applied to 61 matched patients. Meyer et al. (TOPMOST Registry)^17^ included 233 patients with isolated DMVO stroke in the P2/P3 or A2–A4 segments of the PCA and ACA, without PSM. Radu et al. (MAD-MT)^16^ included 1610 patients with medium proximal vessels (M2, A1, P1) and medium distal vessels (M3, A2, P2), with PSM applied to 668 matched patients. Berberich et al. (PLATO Cohort)^30^ included 376 patients who underwent EVT for AIS due to isolated occlusion of the PCA with proximal to distal occlusion sites (P1, P2, P3, or fetal PCA), without PSM. Mehta et al.^28^ included 279 patients with M2, M3, or M4 occlusion; A1 or A2 occlusion; and P1 or P2 occlusion, without PSM. The median age ranged from 68 to 76 years across the studies, with a slightly higher proportion of female patients in the GA group. Hypertension prevalence ranged from 70% to 83.6% in both groups. The median NIHSS scores at baseline were similar between the two groups, indicating comparable stroke severity. The site of occlusion included various segments of the MCA, ACA, and PCA, with some studies focusing on specific segments (Supplemental Table S2).

### Procedural outcomes

#### Unmatched overall results

In the unmatched overall results, the time from stroke onset to groin puncture was numerically higher in the GA group, with a median of 204 min compared to 189 min in the non-GA group (*p* = 0.07). Procedural time was similar between the groups, with a median of 52 min for the GA group and 55 min for the non-GA group (*p* = 0.80). FPE was achieved in 28.3% of the GA group and 27.8% of the non-GA group (*p* = 0.91, Table 1). Adjusted results for the overall cohort indicated that the GA group had higher rates of successful recanalization (71.0% vs 64.7%, aOR 1.60, 95% CI (1.02–2.50), *p* = 0.04), excellent recanalization (54.5% vs 43.8%, aOR 1.82, 95% CI (1.21–2.74), *p* = 0.004), and complete recanalization (45.5% vs 33.8%, aOR 1.92, 95% CI (1.28–2.90), *p* = 0.002, Table 2).

**Table 2.** Outcomes in adjusted analysis of GA versus non-GA in overall and matched cohorts.

Outcomes	Unmatched				Matched			
	*N*	Effect estimate*	a(c)OR, coefficient (β) (95% CI)	*p*-value	*N*	Effect estimate	a(c)OR, coefficient (β) (95% CI)	*p*-value
Procedural outcomes								
FPE	595	aOR	1.04 (0.67–1.64)	0.83	163	OR	1.68 (0.81–3.47)	0.16
mFPE	595	aOR	1.00 (0.66–1.52)	0.99	163	OR	1.23 (0.63–2.83)	0.54
Successful recanalization—*n* (%)	595	aOR	1.60 (1.02–2.50)	0.04	163	OR	2.66 (1.38–5.12)	0.003
Excellent recanalization—*n* (%)	595	aOR	1.82 (1.21–2.74)	0.004	163	OR	3.31 (1.74–6.29)	<0.001
Complete recanalization—*n* (%)	595	aOR	1.92 (1.28–2.90)	0.002	163	OR	2.96 (1.54–5.69)	0.001
Functional outcomes								
NIHSS 24 at 24–48 h	607	Coefficient (β)	0.41 (−1.15–1.97)	0.61	164	Coefficient (β)	0.06 (−1.67–3.74)	0.45
mRS 0–1 or RoR at 90 days^#^	463	aOR	0.68 (0.40–1.16)	0.16	134	OR	0.92 (0.45–1.86)	0.82
mRS 0–2 or RoR at 90 days^#^	511	aOR	1.11 (0.68–1.82)	0.68	170	OR	1.38 (0.72–2.64)	0.33
mRS 0–3 or RoR at 90 days^#^	541	aOR	0.77 (0.47–1.28)	0.32	160	OR	0.85 (0.45–1.59)	0.62
mRS (ordinal shift analysis) at 90 days	565	acOR	0.75 (0.52–1.09)	0.14	170	cOR	0.93 (0.54–1.59)	0.77
Safety outcomes								
Mortality at 90 days	565	aOR	1.49 (0.90–2.47)	0.12	170	OR	1.11 (0.59–2.11)	0.74
sICH—*n* (%)	619	aOR	0.76 (0.34–1.73)	0.52	170	OR	0.42 (0.14–1.27)	0.12

^*^Adjusted for age, NIHSS, ASPECTS, site of occlusion, and premRS, RoR, return of Rankin.

^#^Cases with pre-mRS > mRS outcome and did not return to baseline assessed were excluded.

#### Matched results

In the matched results, the time from stroke onset to groin puncture was 195 min for the GA group and 183 min for the non-GA group (*p* = 0.78), and procedural time was 52 min for the GA group and 55 min for the non-GA group (*p* = 0.80, Table 1). The FPE was achieved in 29.3% of the GA group and 19.8% of the non-GA group (OR 1.68, 95% CI (0.81–3.47), *p* = 0.16). However, the GA group had significantly higher rates of successful recanalization (73.2% vs 50.6%, OR 2.66, 95% CI (1.38–5.12), *p* = 0.003), excellent recanalization (61.0% vs 32.1%, OR 3.31, 95% CI (1.74–6.29), *p* < 0.001), and complete recanalization (52.4% vs 27.2%, OR 2.96, 95% CI (1.54–5.69), *p* = 0.001, Table 2).

#### Meta-analysis

**Figure 1.**
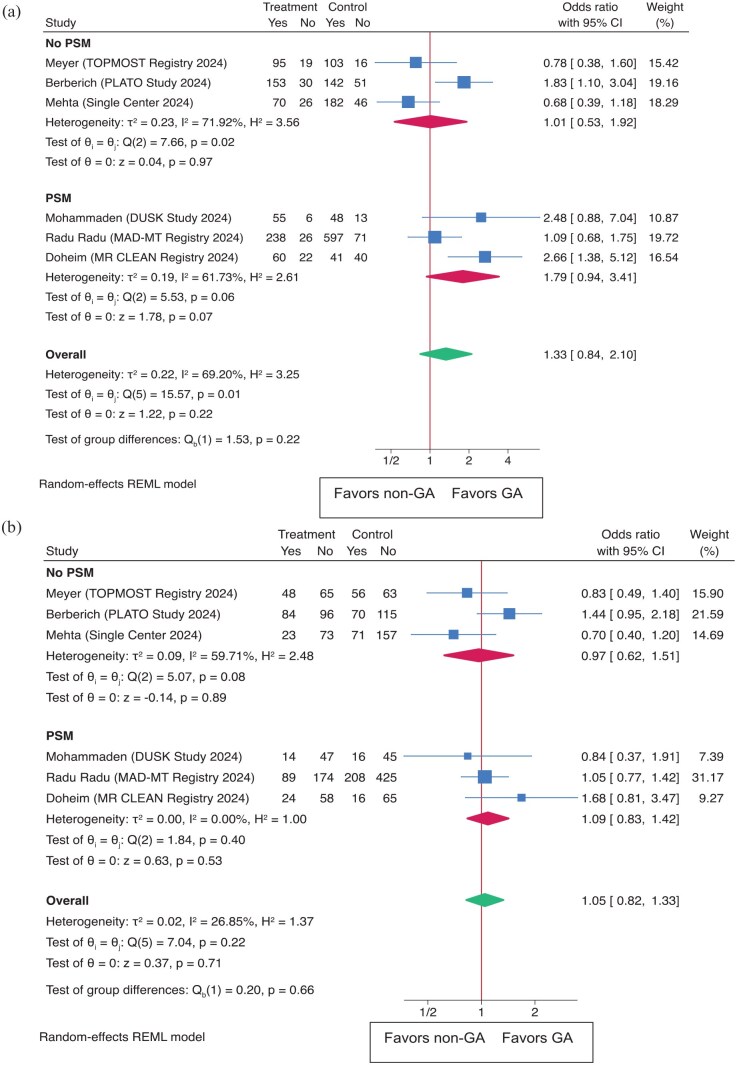
Outcomes as (a) successful recanalization (TICI 2B-3) and (b) first pass effect (FPE) in both the overall population and subgroups based on propensity score matching (PSM).

In the meta-analysis comparing GA to non-GA for procedural outcomes, the overall OR for successful recanalization (TICI 2b-3) was 1.33 (95% CI: 0.84–2.10 (*p* = 0.22); Figure 1(a)). Subgroup analysis revealed that the OR for the PSM group was 1.79 (95% CI: 0.94–3.41), and for the non-PSM group, it was 1.01 (95% CI: 0.53–1.92). For the FPE (TICI 2c-3 with the first pass), the overall OR was 1.05 (95% CI: 0.82–1.33), *p* = 0.72 (Figure 1(b)). In the PSM subgroup, the OR was 1.09 (95% CI: 0.84–1.42), while the non-PSM group showed an OR of 0.97 (95% CI: 0.62–1.51). Leave-one-out analysis yielded consistent results (Supplemental Figure S1).

### Functional outcomes

#### Unmatched overall results

**Figure 2.**
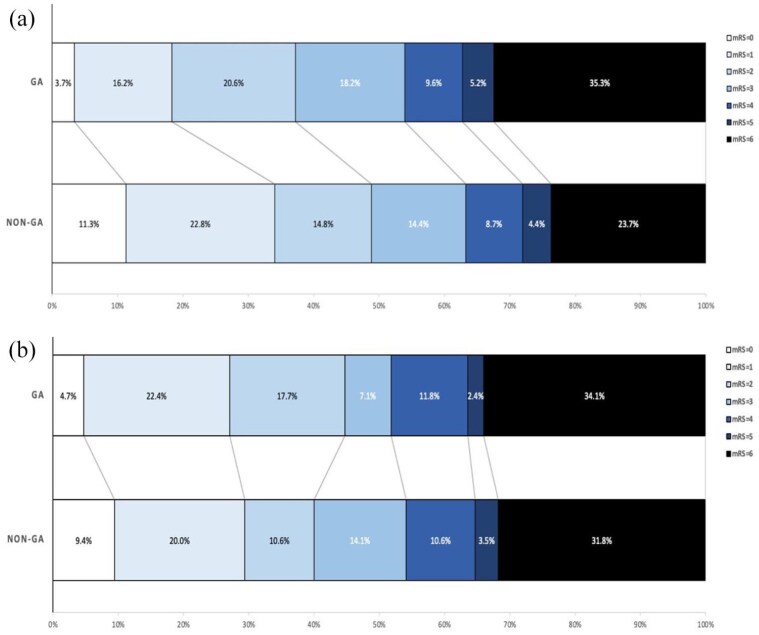
Distribution of modified Rankin scale scores at 90 days in (a) overall and (b) matched.

In the unmatched overall results, patients in the GA group showed worse functional outcomes at 90 days. Specifically, a lower percentage of GA patients achieved an mRS score of 0–1 (19.9% vs 34.1%; *p* = 0.01) and mRS 0–3 (50.0% vs 63.3%; *p* = 0.01). However, the difference in achieving an mRS score of 0–2 was not significant (40.4% vs 48.9%; *p* = 0.08, Table 1). Adjusted overall results showed no significant differences in functional outcomes comparing the GA to the non-GA groups, with adjusted ORs of 0.68 (95% CI: 0.40–1.16) for mRS 0–1 (*p* = 0.16), 1.11 (95% CI: 0.68–1.82) for mRS 0–2 (*p* = 0.68), and 0.77 (95% CI: 0.47–1.28) for mRS 0–3 (*p* = 0.32). Additionally, the acOR for the mRS shift at 90 days was 0.75 (95% CI: 0.52–1.09), *p* = 0.14), indicating no significant difference in the shift toward better mRS scores between the GA and non-GA groups (Figure 2(a), Table 2).

#### Matched results

**Figure 3.**
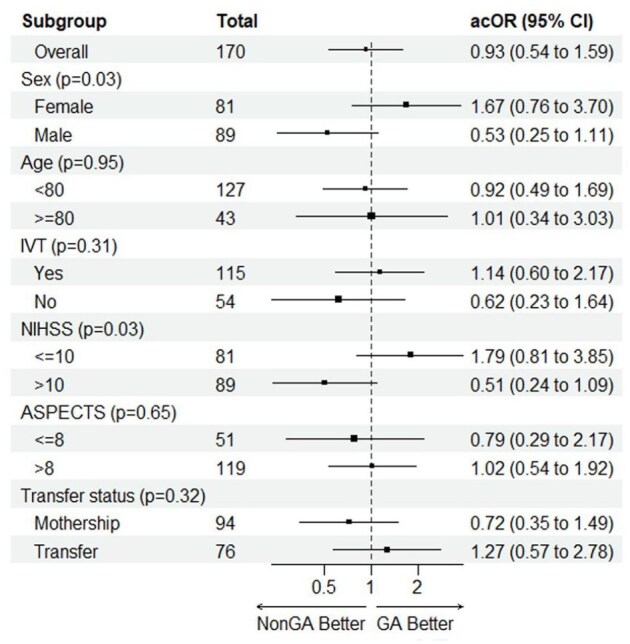
Forest plot showing GA versus non-GA effects for the ordinal shift in the mRS scores at 90 days in pre-specified subgroups from matched MR CLEAN Registry groups. acOR: adjusted common odds ratio; ASPECTS: acute stroke prognosis early CT score; IVT: intravenous thrombolysis; NIHSS: National Institutes of Health Stroke Scale.

In the matched results, there were no significant differences in functional outcomes between the GA and non-GA groups. The percentages of patients achieving mRS scores of 0–1, 0–2, and 0–3 were 27.1% versus 29.4% (OR 0.92 (95% CI: 0.45–1.86), *p* = 0.73), 44.7% versus 40.0% (OR 1.38 (95% CI: 0.72–2.64), *p* = 0.54), and 51.8% versus 54.1% (OR 0.85 (95% CI: 0.45–1.59), *p* = 0.76), respectively (Tables 1 and 2). The cOR for the mRS shift at 90 days in the matched cohort was 0.93 ((95% CI: 0.54–1.59), *p* = 0.77), further indicating no significant difference in the shift toward better mRS scores for GA compared to non-GA groups (Figure 2(b)). In the subgroup interaction analyses of the matched groups (Figure 3), the ordinal shift analysis of mRS scores across various subgroups showed no significant benefit of GA on functional outcomes for age (<80, ⩾80), IVT, ASPECTS (⩽8, >8), and mothership versus transfer subgroups. However, interaction analysis was positive for NIHSS, showing that cases with NIHSS ⩽10 may benefit from GA more than cases with NIHSS >10 (*p*_interaction_ = 0.03). The same was noticed for the sex subgroup interaction analysis, showing that females benefited more from GA than males, with *p*_interaction_ = 0.03.

#### Meta-analysis

**Figure 4.**
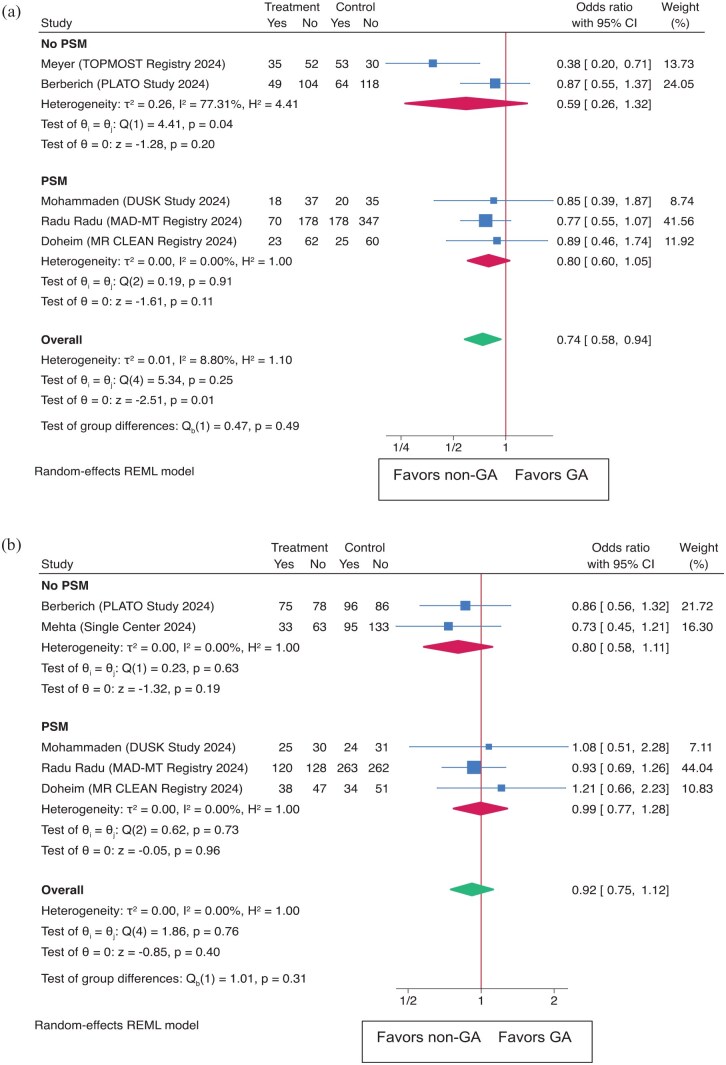
shows functional outcomes as (a) excellent functional outcome (mRS score of 0–1 at 90 days) and (b) functional Independence (mRS score of 0–2 at 90 days) in both the overall population and subgroups based on propensity score matching (PSM).

In a meta-analysis of functional outcomes, comparing GA to non-GA for achieving mRS 0–1 at 90 days, the overall OR was 0.74 (95% CI: 0.58–0.94), with a significant *p*-value (*p* = 0.01, Figure 4(a)). The PSM subgroup had an OR of 0.80 (95% CI: 0.60–1.05), while the non-PSM group had an OR of 0.59 (95% CI: 0.26–1.32). The leave-one-out analysis (Supplemental Figure S1) confirmed the robustness of the results. For mRS 0–2 at 90 days, the overall OR was 0.92 (95% CI: 0.75–1.12), *p* = 0.40 (Figure 4(b)). In the PSM subgroup, the OR was 0.99 (95% CI: 0.77–1.28), and in the non-PSM group, the OR was 0.80 (95% CI: 0.58–1.11). The leave-one-out analysis (Supplemental Figure S1) did not significantly alter these results.

### Safety outcomes

#### Unmatched overall results

In the unmatched overall results, mortality at 90 days was significantly higher in the GA group compared to the non-GA group (35.3% vs 23.7%; *p* = 0.01). However, there were no significant differences in the rates of sICH (6.0% vs 7.1%; *p* = 0.62) and pneumonia (10.6% vs 10.5%; *p* = 0.99) between the GA and non-GA groups (Table 1). Adjusted overall results showed no significant differences in mortality at 90 days (adjusted OR 1.49 (95% CI: 0.90–2.47), *p* = 0.12) and sICH (adjusted OR 0.76 (95% CI: 0.34–1.73), *p* = 0.52, Table 2).

#### Matched results

In the matched results, mortality at 90 days was similar between the GA and non-GA groups (34.1% vs 31.8%; OR 1.11 (95% CI: 0.59–2.11), *p* = 0.74). There were also no significant differences in the rates of sICH (5.9% vs 12.9%; OR 0.42 (95% CI: 0.14–1.27), *p* = 0.12) and pneumonia (15.3% vs 21.2%; *p* = 0.32) between the GA and non-GA groups. These findings indicate that while mortality was initially higher in the GA group, this difference was not observed after matching, and there were no significant differences in other safety outcomes between the two groups (Tables 1 and 2).

#### Meta-analysis

**Figure 5.**
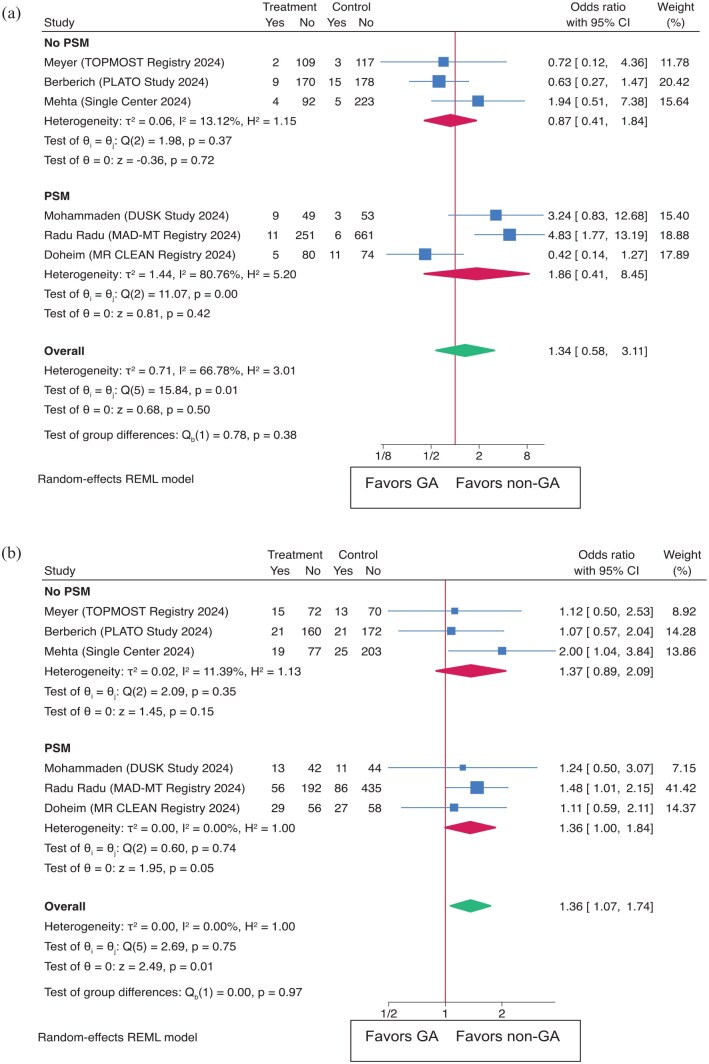
Outcomes as (a) sICH (symptomatic intracerebral hemorrhage) and (b) Mortality at 90 days in both the overall population and subgroups based on propensity score matching (PSM).

This meta-analysis also examined safety outcomes, comparing GA to non-GA for sICH. The overall OR was 1.34 (95% CI: 0.58–3.11 (*p* = 0.50)) (Figure 5(a)). The PSM subgroup had an OR of 1.86 (95% CI: 0.41–8.46), while the non-PSM group had an OR of 0.87 (95% CI: 0.41–1.84). For 90-day mortality (Figure 5(b)), the overall OR was 1.36 (95% CI: 1.07–1.74 (*p* = 0.01)). Subgroup analysis revealed an OR of 1.36 (95% CI: 1.00–1.84) in the PSM group and 1.37 (95% CI: 0.90–2.09) in the non-PSM group. The leave-one-out analysis confirmed the significance of these findings for sICH and mortality in 90 days (Supplemental Figure S1).

## Discussion

Our comprehensive analysis, which included a PSM cohort from the MR CLEAN registry and a meta-analysis of six studies, revealed insights into the impact of anesthesia modality on procedural, and clinical outcomes in patients undergoing EVT for DMVOs. The higher rates of recanalization observed with GA in the MR CLEAN registry suggest that GA may offer technical advantages, possibly due to better patient immobility and improved imaging quality, facilitating precise clot retrieval. However, the absence of significant differences in the FPE between GA and non-GA in both the MR CLEAN registry and the pooled meta-analysis indicates that while GA may improve recanalization success, it does not necessarily reduce the number of passes required for achieving this outcome, pointing to no clear enhancement in procedural efficiency with GA. Likewise, despite the procedural benefits associated with GA, our primary analysis of MR CLEAN registry found no significant differences in functional outcomes at 90 days between the GA and non-GA groups after matching. Additionally, the meta-analysis revealed higher mortality rates and fewer excellent outcomes (mRS 0–1) associated with GA. This highlights a critical consideration for clinical practice: while GA may improve recanalization rates, it does not independently drive better long-term functional recovery.^5,7,10,13^ Functional outcomes, such as mRS scores, are influenced by a plethora of factors beyond recanalization success, including peri-procedural hemodynamic stability, sedation effects on cerebral perfusion, and patient-specific factors like baseline stroke severity and comorbidities.^5,13^

Two recent trials, DISTAL and ESCAPE-MeVO, assessed EVT for DMVOs did not demonstrate a significant reduction in overall disability, or mortality compared to best medical management.^20,21^ Both trials reported a median NIHSS of 6–8, suggesting that patients with mild to moderate strokes were overrepresented, raising concerns about potential selection bias. Additionally, ESCAPE-MeVO reported a higher incidence of serious adverse events, such as pneumonia and infections, which may have been influenced by procedural sedation. Another factor that may have contributed to the lack of benefit in these trials was treatment delay, with a median onset-to-recanalization time of 359 min in ESCAPE-MeVO compared to 241 min in ESCAPE, potentially reducing the volume of salvageable tissue.^22^ This can be explained by the heavy use of GA in this group of patients and should be carefully considered in future studies.^20,21^

A key consideration in our primary analysis of the MR CLEAN Registry is the anatomical heterogeneity within M2 occlusions, which complicates their classification and clinical interpretation. While this cohort consisted almost exclusively of M2 occlusions (>95%), detailed information on occlusion dominance, branch involvement, or proximal versus distal location was unavailable for this analysis. Many of these cases may have involved proximal, dominant M2 branches, which are not universally categorized as distal vessel occlusions, limiting the generalizability of findings to true distal occlusions. In a multicenter retrospective study that evaluated the impact of GA versus non-GA on outcomes in patients with minor stroke and isolated M2 occlusion undergoing MT, no significant differences were found between groups in 90-day functional outcomes, mortality, successful reperfusion, or procedure-related complications.^33^ Notably, the DISTAL trial specifically included occlusions of nondominant or codominant M2 segments, whereas ESCAPE-MeVO enrolled patients with proximal M2 occlusions in a significant proportion of cases (64/253 (25.3%) in EVT versus 58/269 (21.6%) in the control group), raising the question of whether proximal M2 occlusions may represent medium or even large vessel occlusions in certain contexts. Nonetheless, understanding the optimal anesthesia strategy in this anatomically diverse group remains clinically valuable.^18,20,21^

In our main analysis of the MR CLEAN registry and the subsequent meta-analysis, no significant differences in sICH were observed between the groups after matching. This absence of differences indicates that either GA or non-GA can be utilized safely without markedly increasing the risk of complications, a crucial factor in clinical decision-making. Notably, non-GA was associated with reduced mortality compared to GA, suggesting potential benefits beyond procedural safety. While the use of GA may be favored in cases where its technical advantages are critical for achieving successful recanalization, particularly in complex cases involving fragile distal vessels, non-GA strategies remain safe, and effective. Moreover, they may reduce risks such as peri-procedural hypotension, making non-GA a viable option in many clinical scenarios.^10,13,19,34^

Our primary analysis of MR CLEAN registry highlights the importance of individualized anesthesia strategies in EVT for DMVOs. Non-GA strategies, which may offer similar functional recovery outcomes with reduced risks, should be considered in many cases.^5,10,34^ One particularly noteworthy finding from our interaction analysis is the apparent greater benefit of GA in patients presenting with lower NIHSS scores—a result that may initially seem counterintuitive. Conventionally, patients with milder strokes are often selected for non-GA approaches, under the assumption that they will better tolerate the procedure and that avoiding intubation reduces risk. However, our findings suggest that GA may be beneficial even in this subgroup. A potential explanation lies in the lower recanalization rates observed in the non-GA group, which could disproportionately affect patients with lower baseline deficits. In such patients, failure to achieve recanalization may lead to substantial neurological worsening, outweighing any advantage of avoiding GA. Moreover, GA may provide a more controlled procedural environment, minimizing patient movement and optimizing imaging and catheter manipulation—factors that may be particularly critical in technically challenging MeVO procedures. These observations align with recent evidence study which compared best medical management to unsuccessful EVT in patients with M2 and M3 occlusions. In their propensity-matched analysis of 532 patients, medical management was associated with significantly better functional outcomes (mRS 0–1 in 32% vs 21%, *p* = 0.011), lower rates of symptomatic ICH (3.4% vs 16%, *p* < 0.001), and fewer hemorrhagic complications overall. Importantly, unsuccessful MT was linked to worse functional outcomes and higher odds of hemorrhage, reinforcing the idea that procedural failure—potentially more frequent in non-GA settings—may lead to harm. Together, these findings urge the need for careful anesthesia planning, even in patients with lower NIHSS, and highlight the risks associated with suboptimal recanalization.^35^ We fully acknowledge that these findings from interaction analyses are exploratory and hypothesis-generating rather than conclusive. Although the mRS shift analysis was neutral after propensity score matching, the proportion of patients achieving mRS 0–2 was numerically higher in the matched GA group. Adequate matching in our primary cohort appears to mitigate the initial trend toward unfavorable outcomes with GA, which was likely influenced by baseline imbalances favoring the non-GA group.

Several limitations should be acknowledged. This study was retrospective, and despite PSM to balance baseline characteristics, residual confounding cannot be completely ruled out. Additionally, the majority of patients included in our primary analysis of the MR CLEAN registry had MCA-M2 occlusions, with a smaller number of patients having MCA-M3 and ACA-A1/A2 occlusions, and we excluded posterior circulation DMVOs (PCAs) to avoid potential selection bias among other reported limitations.^25^ We could not distinguish between dominant/nondominant or proximal/distal M2 occlusions, but it is expected that we included proximal/dominant M2 occlusions. However, we enhanced our study through conducting a pooled analysis with five additional studies, which included a diverse set of lesions beyond MCA-M2, such as MCA-M3/M4, ACA-A1/2/3, and PCA P1/P2/3. Early analyses from the MR CLEAN registry (patients treated between March 2014 and June 2016) showed no significant difference in neurological recovery between dominant (*n* = 124) and co/non-dominant M2 (*n* = 51) occlusions (mean ΔNIHSS: −2 ± 10 vs −5 ± 5, respectively). Moreover, the effect of reperfusion on functional outcomes was comparable (common OR for good outcome: 1.27 (95% CI: 1.06–1.53) for dominant M2 vs 1.32 (95% CI: 0.93–1.87) for nondominant M2).^36^ Similarly, in the HERMES meta-analysis, 130 patients with M2 occlusions were included, with breakdowns across proximal (*n* = 116) and distal (*n* = 14) locations, anterior (*n* = 72) versus posterior (*n* = 58) divisions, and dominant (*n* = 73), codominant (*n* = 50), and nondominant (*n* = 7) branches—further illustrating the complexity and variability within M2 occlusion classification.^37^ Another limitation of this analysis is the variability in anesthetic management practices across hospitals. Specifically, we were unable to account for the type and dose of anesthetic used (e.g. propofol, a potent cerebral vasoconstrictor, vs sevoflurane, a cerebral vasodilator), nor were we able to assess how end-tidal or arterial CO_2_ (ETCO_2_/PaCO_2_) levels and blood pressure (MAP or SBP) were managed during the procedure. Specifically, we were unable to account for the type and dose of anesthetic used (e.g. propofol, a potent cerebral vasoconstrictor, vs sevoflurane, a cerebral vasodilator), nor were we able to assess how end-tidal or arterial CO_2_ (ETCO_2_/PaCO_2_) levels and blood pressure (MAP or SBP) were managed during the procedure. These variables are known to significantly affect cerebral perfusion, particularly in the context of ischemia, and could introduce confounding or bias in interpreting the effect of general anesthesia versus non-general anesthesia. While propensity score matching was employed to adjust for observable baseline imbalances, unmeasured factors such as anesthetic technique and physiological management during thrombectomy remain unaccounted for. We acknowledge that the lack of anesthetic expertise in the study team may have limited the depth of reporting and interpretation regarding the complexity of GA as an intervention. Future work should incorporate academic anesthetist input to better capture relevant anesthesia-related parameters. Future prospective studies with standardized anesthetic protocols and detailed intraoperative monitoring are needed to clarify the role of these variables and to better understand how anesthesia modality interacts with cerebral hemodynamics in the setting of acute ischemic stroke. We did not include a random effects factor for hospitals due to challenges with model convergence, likely stemming from sample size limitations within hospitals, potential overfitting risks with sparse data, and the complex variance structure of anesthetic practices across sites. Previous analysis of MR CLEAN registry data confirmed that functional outcome was similar after adjustment for center in a multilevel regression analysis considering different centers.^11^ These factors could impact our ability to accurately estimate hospital-level effects and should be considered when interpreting our findings. While subgroup analyses of the primary analysis suggest that certain patients may benefit more from GA, these findings should be considered exploratory and warrant validation in future prospective trials. In our meta-analysis, we observed significant heterogeneity for some outcomes that could not be resolved through sensitivity analysis. However, we addressed this by using a random effects model to account for the heterogeneity. Study-level data prevented subgroup interaction analyses such as anterior versus posterior and proximal medium versus distal vessel occlusions. The primary analysis was conducted as an as-treated analysis, which better reflects real-world clinical decision-making regarding anesthesia strategy during EVT. We acknowledge that a one-way crossover from non-GA to GA may introduce bias, particularly since patients requiring conversion are often those who experience agitation, clinical deterioration, or procedural complications—factors independently associated with poorer outcomes. Although conversion data were not available in the primary registry, prior analyses from the MR CLEAN Registry reported a conversion rate of approximately 8%.^11^ In our meta-analysis, two studies provided conversion data, ranging from 2% to 6%.^28,30^ This remains an important source of bias and highlights the need for standardized reporting of conversion events in future research. Additionally, the relatively small number of patients in our study, along with the limited number of available studies for pooled analysis, precluded assessment of publication bias or the performance of meta-regression analyses. The population of the included studies may have overlapped, as there were multicenter studies conducted across overlapping periods. We did not have access to patient-level data to confirm and exclude any overlapping patients. We tabulated the results for each study to overcome this limitation (Supplemental Table S3). Furthermore, some included studies did not employ PSM, though we conducted subgroup analyses categorizing the studies into those with and without PSM, in addition to performing leave-one-out analyses to strengthen our findings. The meta-analysis comprised studies with varying designs, periods, and eligibility criteria, which may introduce heterogeneity into the results. Importantly, anesthesia practices and procedural protocols differ between centers, potentially influencing outcomes.

### Conclusion

Our PSM analysis of the MR CLEAN registry, combined with our meta-analysis, highlights the nuanced relationship between anesthesia modality, and outcomes in EVT for DMVOs. While GA may improve technical outcomes related to recanalization, these procedural gains do not necessarily translate into better functional recovery or lower mortality at 90 days. Additional meta-analysis showed that non-GA strategies were associated with improved functional recovery, evidenced by higher rates of excellent outcomes and reduced mortality at 90 days. These associations, however, warrant cautious interpretation given potential unmeasured confounders, including blood pressure management and conversion from non-GA to GA. Broad categorization of anesthesia as GA versus non-GA overlooks critical factors such as agent selection, physiological targets, and intraoperative monitoring, which may substantially impact cerebral perfusion and outcomes. Further prospective randomized studies with detailed anesthetic data and expert input are needed to validate our findings and confirm their clinical relevance across diverse DMVO subgroups.

## Supplementary Material

sj-docx-1-eso_23969873251352406

## Data Availability

Data cannot be made available to other researchers, as no patient approval has been obtained for sharing coded data. However, syntax and output files of statistical analyses are available on reasonable request.

## Nonstandard abbreviations and acronyms

aOR adjusted odds ratio

CT computed tomography

TICI Thrombolysis in Cerebral Ischemia

EVT endovascular treatment

IVT intravenous thrombolysis

LVO large vessel occlusion

DMVO Distal or medium vessel occlusion

mRS modified Rankin Scale

sICH symptomatic intracranial hemorrhage
