# Supplementary material for: Association of anesthesia strategies with outcomes in endovascular treatment for distal and medium vessel occlusions: A propensity score-matched analysis of the MR CLEAN registry and meta-analysis
Source: Eur Stroke J. 2026 Jan 1;11(1):23969873251352406. doi: 10.1093/esj/23969873251352406 (PMC12866256; doi:10.1093/esj/23969873251352406)
Supplement: sj-docx-1-eso_23969873251352406 [file sj-docx-1-eso_23969873251352406.docx]

***Supplements for “Anesthesia Strategies in Endovascular Treatment for Distal or Medium Vessel Occlusions: Propensity Score Matched Analysis of the MR CLEAN Registry and a Systematic Review and Meta-Analysis of Six Studies”***

**Table S1: Summary of the included studies**

| First author, year | Study design | Study Period | Eligibility | PSM | Total sample (GA: non-GA) | PSM sample |
| --- | --- | --- | --- | --- | --- | --- |
| Mohammaden (DUSK Cohort) 2024 | Multicenter retrospective analysis | January 2017 to June 2021 | DMVO involving the middle cerebral artery-M3/4, anterior cerebral artery-A2/3, or posterior cerebral artery-P1/P2-3, and underwent EVT | Yes | 366 (67:299) | 61 pairs |
| Meyer (TOPMOST Registry) 2024 | Multicenter retrospective analysis | January 1, 2010, and October 30, 2021 | Isolated DMVO stroke: Occlusions were in the P2/P3 or A2–A4 segments of the posterior and anterior cerebral arteries (PCA and ACA) | No | 233 (114:119) | NA |
| Radu (MAD-MT) 2024 | Multicenter retrospective analysis | September 2016 and December 2021 | Medium proximal vessels: M2, A1, P1, and medium distal vessels: M3, A2, P2, and distal. | Yes | 1610 (395:1215) | 264 (GA):668 (Non-GA) |
| Berberich (PLATO Cohort) 2024 | Multicenter retrospective analysis | January 2015 and August 2022 | EVT for acute ischemic stroke due to isolated occlusion of the PCA with proximal to distal occlusion sites (P1, P2, P3 or fetal PCA) within 24 hours after symptom onset and prestroke modified Rankin scale (mRS) score of 0 to 3 | No | 376 (183:193) | NA |
| Mehta 2024 | Retrospective single center | December 2014 to July 2023 | M2, M3, or M4 occlusion; A1 or A2 occlusion; and P1 or P2 occlusion | No | 279 (69:193) | NA |
| Doheim (MR CLEAN Registry) 2024 | Multicenter retrospective analysis | March 2014 and December 2018 | Anterior distal or medium vessel occlusion: MCA: M2, M3, ACA: A1, A2 | Yes | 657 (151:506) | 85 pairs |

**Table S2. Baseline characteristics of the included studies**

| First author, year | Group | Age | Female | Hypertension | DM | AF | LKW to puncture time | NIHSS at baseline | Site of occlusion |
| --- | --- | --- | --- | --- | --- | --- | --- | --- | --- |
| Mohammaden (DUSK Cohort) 2024 | GA | 69 (60–82) | 28 (41.8%) | 55 (82.1%) | 24 (35.8%) | 25 (37.3%) | 327.5 (236–668) | 15 (9–24) | MCA-M3: 25 (37.3%)  MCA-M4: 2 (3%)  ACA-A2/3: 4 (6%)  PCA-P1: 22 (32.8%)  PCA-P2/3: 14 (20.9%) |
|  | Non-GA | 69 (61–80) | 116 (38.8%) | 238 (79.6%) | 98 (32.8%) | 96 (32.1%) | 317 (198–640) | 10 (7–16) | MCA-M3: 25 124 (41.5%) MCA-M4: 6 (2%)  ACA-A2/3: 45 (15.1%)  PCA-P1: 73 (24.4%)  PCA-P2/3: 51 (17.1%) |
|  | GA (PSM) | 69 (59.5–82) | 26 (42.6%) | 51 (83.6%) | 21 (34.4%) | 24 (39.3%) | 330.5 (222–661.9) | 14 (9–21) | MCA-M3: 24 (39.3%)  MCA-M4: 2 (3.3%)  ACA-A2/3: 4 (6.6%)  PCA-P1: 19 (31.1%)  PCA-P2/3: 12 (19.7%) |
|  | Non-GA (PSM) | 68 (62–78.5) | 22 (36.1%) | 51 (83.6%) | 24 (39.3%) | 22 (36.1%) | 267 (183.5–511.5) | 14 (8–21) | MCA-M3: 20 (32.8%)  MCA-M4: 0 (0%)  ACA-A2/3: 7 (11.5%)  PCA-P1: 25 (41%)  PCA-P2/3: 9 (14.8%) |
| Meyer (TOPMOST Registry) 2024 | GA | 76 (64–82) | 52.1 (62) | 79.8 (91) | 22.8 (26) | 43 (49) | 190 (147–294) | 9 (5–14) | ACA: 47.4 (54) PCA: 52.6 (60) Distal (segments of A3, A4 or P3): 23.7 (27) |
|  | Non-GA | 75 (64–83) | 50.6 (118) | 73.1 (87) | 18.5 (22) | 36.1 (43) | 197 (135–310) | 7 (4–12) | ACA 33.6 (40) PCA 66.4 (79) Distal=segments of A3, A4 or P3 27.7 (33) |
| Radu (MAD-MT) 2024 | GA | 72 ± 13.4 | 215 (54.4%) | 296 (74.9%) | 95 (24.1%) | 148 (37.5%) | 297 (280–318) | 13 (13–15) | Medium vessel: 318 (80.5%) Distal vessel: 77 (19.5%) |
|  | Non-GA | 73.5 ± 13.2 | 628 (51.7%) | 851 (70%) | 260 (21.4%) | 403 (33.2%) | 335 (321–350) | 11 (11–13) | Medium vessel:994 (81.8%)  Distal vessel: 221 (18.2%) |
|  | GA (PSM) | 72.1 ± 13.6 | 136 (51.5%) | 191 (72.3%) | 59 (22.3%) | 117 (44.3%) | 301 (283–325) | 13 (12–15) | Medium vessel: 222 (84.1%) Distal vessel: 42 (15.9%) |
|  | Non-GA (PSM) | 73 ± 13.2 | 363 (54.3%) | 489 (73.2%) | 167 (25%) | 292 (43.7%) | 321 (303–343) | 13 (13–14) | Medium vessel: 565 (84.6%) Distal vessel 103 (15.4%) |
| Berberich (PLATO Cohort) 2024 | GA | 74 (65–82) | 91 (49.7%) | 142 (77.6%) | 41 (22.4%) | 66 (36.1%) | 3.5 (2.3–7) | 8 (5–14) | P1 or fetal PCA: 97 (53.0)  P2:77 (42.1)  P3: 12 9 (4.9) |
|  | Non-GA | 73 (63–81) | 72 (37.3%) | 146 (75.7%) | 45 (23.3%) | 57 (29.5%) | 3.9 (2.4–7.9) | 8 (5–12) | P1 or fetal PCA: 116 (60.1)  P2: 74 (38.3)  P3: 3 (1.6) |
| Mehta 2024 | GA | 73 (58- 78) | 104 (49.52%) | 68 (80.95%) | 29 (34.94%) | 32 (39.51%) | 282.5 (202, 672.75) | 15.5 (10, 20) | Left MCA: 45 (62.5%)  Right MCA: 27 (37.5%)  M2 occlusion: 66 (76.74%)  M3 occlusion: 6 (6.98%) |
|  | Non-GA | 74 (58-78) | 20 (38.37%) | 158 (78.61%) | 64 (31.68%) | 69 (36.32%) | 292 (202, 672.75) | 11 (10, 20) | Left MCA: 118 (64.84%)  Right MCA: 64 (35.16%)  M2 occlusion: 171 (81.43%) M3 occlusion: 11 (5.24%) |
| Doheim (MR CLEAN Registry) 2024 | GA | 73 [61-80] | 65 (43.0%) | 84/150 (55%) | 28 (18.5%) | 132/497 (26.6%) | 204 [157-254] | 13 [9.0-17.0] | M2: 143 (94.7) M3: 5 (3.3)  A1: 2 (1.3)  A2: 1 (0.66) |
|  | Non-GA | 73 [64-81] | 252 (49.8%) | 281/492 (57.1%) | 89/503 (17.7%) | 40/150 (26.7%) | 189 [131-253] | 10 [6.0-16.0] | M2 496 (98.0) M3 3 (0.6) A1 2 (0.4) A2: 5 (0.99) |
|  | GA (PSM) | 74 [66-80] | 37 (43.5%) | 50/84 (59.5%) | 17/85 (20.0%) | 26/85 (30.6%) | 195 [157-245] | 12 [8-16] | M2: 81 (95.3) M3: 3 (3.5)  A1: 0 (0.0)  A2: 0 (0.0) |
|  | Non-GA (PSM) | 72 [63-81] | 44 (51.8%) | 41/80 (51.3%) | 14/83 (16.9%) | 23/83 (27.7%) | 183 [146-256] | 10 [7-16] | M2: 82 (96.5)  M3: 0 (0.0)  A1: 1 (1.2)  A2: (2.4) |

**Table S3. Characteristics and Main Findings of Included Studies Comparing General Anesthesia (GA) vs Non-GA in Posterior Circulation EVT**

| Study | Main Results |
| --- | --- |
| Mohammaden (DUSK Cohort), 2024 | **N=366 (GA: 67 / Non-GA: 299)  • 90-day mRS 0–2: GA 45.5% vs Non-GA 43.6%  • eTICI 2b-3: GA 90.2% vs Non-GA 78.7%  • sICH: GA 15.5% vs Non-GA 4.5%  • 90-day Mortality: GA 23.6% vs Non-GA 20.0%** |
| Meyer (TOPMOST Registry), 2024 | **N=233 (GA: 114 / Non-GA: 119)  • 90-day mRS 0–1: GA 40.2% vs Non-GA 63.9%  • mTICI 2b-3: GA 83.3% vs Non-GA 86.6%  • sICH: GA 1.8% vs Non-GA 2.5%  • 90-day Mortality: GA 17.3% vs Non-GA 15.7%** |
| Radu (MAD-MT), 2024 | **N=1610 (GA: 395 / Non-GA: 1215)  • 90-day mRS 0–2: GA 48.4% vs Non-GA 50.1%  • mTICI 2b-3: GA 90.2% vs Non-GA 89.4%  • sICH: GA 4.2% vs Non-GA 0.9%  • 90-day Mortality: GA 22.6% vs Non-GA 16.5%** |
| Berberich (PLATO Cohort), 2024 | **N=376 (GA: 183 / Non-GA: 193); Conversion rate 2%  • 90-day mRS 0–2: GA 49.0% vs Non-GA 52.8%  • TICI 2b-3: GA 83.6% vs Non-GA 73.6%  • sICH: GA 13.5% vs Non-GA 2.2%  • 90-day Mortality: GA 5.0% vs Non-GA 7.9%** |
| Mehta, 2024 | **N=262 (GA: 69 / Non-GA: 193); Conversion rate 6%  • 90-day mRS 0–3: GA 34.4% vs Non-GA 41.7%  • TICI 2b-3: GA 72.9% vs Non-GA 79.8%  • ICH: GA 4.2% vs Non-GA 2.2%  • 90-day Mortality: GA 19.8% vs Non-GA 11.0%  • Neurological improvement: GA 72.9% vs Non-GA 79.8%** |
| Doheim (MR CLEAN Registry), 2024 | **N=170 (GA: 85 / Non-GA: 85)  • Time to groin puncture: 195 [157–245] vs 183 [146–256] min (p=0.78)  • Procedure time: 52 [31–82] vs 55 [36–70] min (p=0.80)  • FPE: 29.3% vs 19.8% (p=0.16)  • mFPE: 34.2% vs 29.6% (p=0.56)  • Successful recanalization: 73.2% vs 50.6% (p=0.003)  • Excellent recanalization: 61.0% vs 32.1% (p<0.001)  • Complete recanalization: 52.4% vs 27.2% (p=0.001)  • Vessel dissection: 1.3% vs 2.5% (p=0.57)  • Perforation: 0.0% vs 4.9% (p=0.12)  • Embolization in new territories: 3.8% vs 1.2% (p=0.30)  • NIHSS at 24–48h: 8 [4–16] vs 8 [3–15] (p=0.43)  • mRS 0–1: 27.1% vs 29.4% (p=0.73)  • mRS 0–2: 44.7% vs 40.0% (p=0.54)  • mRS 0–3: 51.8% vs 54.1% (p=0.76)  • Mortality: 34.1% vs 31.8% (p=0.74)  • sICH: 5.9% vs 12.9% (p=0.12)  • Pneumonia: 15.3% vs 21.2% (p=0.32)** |

**A) mRS 0-1:**

**B) mRS0-2:**

**C) sICH**

**D) Mortality**

**E) TICI2B-3**

**F) FPE**

**Figure S1 illustrates the leave-one-out analysis for the following outcomes: A) Excellent Functional Outcome (mRS score of 0–1), B) Functional Independence (mRS score of 0–2), C) sICH (symptomatic intracerebral hemorrhage), D) Mortality, E) Successful Recanalization (TICI 2B-3), and F) First Pass Effect (FPE).**

**MR CLEAN Registry investigators:**

**Executive committee**

Diederik W.J. Dippel^1^; Aad van der Lugt^2^; Charles B.L.M. Majoie^3^; Yvo B.W.E.M. Roos^4^; Robert J. van Oostenbrugge^5,44^; Wim H. van Zwam^6,44^; Jelis Boiten^14^; Jan Albert Vos^8^

**Study coordinators**

Ivo G.H. Jansen^3^; Maxim J.H.L. Mulder^1,2^; Robert- Jan B. Goldhoorn^5,6,44^; Kars C.J. Compagne^2^; Manon Kappelhof^3^; Josje Brouwer^4^; Sanne J. den Hartog^1,2,40^; Wouter H. Hinsenveld ^5,6^

**Local principal investigators**

Diederik W.J. Dippel^1^; Bob Roozenbeek^1^; Aad van der Lugt^2^; Pieter Jan van Doormaal^2^, Charles B.L.M. Majoie^3^; Yvo B.W.E.M. Roos^4^; Bart J. Emmer^3^; Jonathan M. Coutinho^4^; Wouter J. Schonewille^7^; Jan Albert Vos^8^; Marieke J.H. Wermer^9^; Marianne A.A. van Walderveen^10^; Adriaan C.G.M. van Es^10^; Julie Staals^5,44^; Robert J. van Oostenbrugge^5,44^; Wim H. van Zwam^6,44^; Pieter-Jan van Doormaal^2^, Jeannette Hofmeijer^11^; Jasper M. Martens^12^; Geert J. Lycklama à Nijeholt^13^; Jelis Boiten^14^; Sebastiaan F. de Bruijn^15^; Lukas C. van Dijk^16^; H. Bart van der Worp^17^; Rob H. Lo^18^; Ewoud J. van Dijk^19^; Hieronymus D. Boogaarts^20^; J. de Vries^22^; Paul L.M. de Kort^21^; Julia van Tuijl^21^; Issam Boukrab^26^; Jo P. Peluso^26^; Jan S.P. van den Berg^22^; Heleen M. den Hertog^22^; Boudewijn A.A.M. van Hasselt^23^; Leo A.M. Aerden^24^; René J. Dallinga^25^; Maarten Uyttenboogaart^28^; Reinoud P.H. Bokkers^29^; Tobien H.C.M.L. Schreuder^30^; Roel J.J. Heijboer^31^; Koos Keizer^32^; Rob A.R. Gons^32^; Lonneke S.F. Yo^33^; Emiel J.C. Sturm^35^; Tomas Bulut^35^; Paul J.A.M. Brouwers^34^; Anouk D. Rozeman^42^; Otto Elgersma^42^, Michel J.M. Remmers^43^; Thijs E.A.M. de Jong^43^.

**Imaging assessment committee**

Charles B.L.M. Majoie^3^(chair); Aad van der Lugt^2^ (chair); Wim H. van Zwam^6,44^; Geert J. Lycklama à Nijeholt^13^; Marianne A.A. van Walderveen^10^; Marieke E.S. Sprengers^3^; Sjoerd F.M. Jenniskens^27^; René van den Berg^3^; Albert J. Yoo^38^; Ludo F.M. Beenen^3^; Alida A. Postma^6.45^; Stefan D. Roosendaal^3^; Bas F.W. van der Kallen^13^; Ido R. van den Wijngaard^13^; Adriaan C.G.M. van Es^10^; Bart J. Emmer^,3^; Jasper M. Martens^12^; Lonneke S.F. Yo^33^; Jan Albert Vos^8^; Joost Bot^36^; Pieter-Jan van Doormaal^2^; Anton Meijer^27^; Elyas Ghariq^13^; Reinoud P.H. Bokkers^29^; Marc P. van Proosdij^37^; G. Menno Krietemeijer^33^; Jo P. Peluso^26^; Hieronymus D. Boogaarts^20^; Rob Lo^18^;Wouter Dinkelaar^41^; Auke P.A. Appelman^29^; Bas Hammer^16^; Sjoert Pegge^27^; Anouk van der Hoorn^29^; Saman Vinke^20^; Sandra Cornelissen^2^; Christiaan van der Leij^6^; Rutger Brans^6^; Jeanette Bakker^41^; Maarten Uyttenboogaart^28^; Miou Koopman^3^; Lucas Smagge^2^; Olvert A. Berkhemer^1,3,6^; Jeroen Markenstein^3^; Eef Hendriks^3^; Patrick Brouwer^10^

**Writing committee**

Diederik W.J. Dippel^1^(chair); Aad van der Lugt^2^; Charles B.L.M. Majoie^3^; Yvo B.W.E.M. Roos^4^; Robert J. van Oostenbrugge^5,44^; Wim H. van Zwam^6,44^; Geert J. Lycklama à Nijeholt^13^; Jelis Boiten^14^; Jan Albert Vos^8^; Wouter J. Schonewille^7^; Jeannette Hofmeijer^11^; Jasper M. Martens^12^; H. Bart van der Worp^17^; Rob H. Lo^18^

**Adverse event committee**

Robert J. van Oostenbrugge^5,44^(chair); Jeannette Hofmeijer^11^; H. Zwenneke Flach^23^

**Trial methodologist**

Hester F. Lingsma^40^

**Research nurses/local trial coordinators**

Naziha el Ghannouti^1^; Martin Sterrenberg^1^; Wilma Pellikaan^7^; Rita Sprengers^4^; Marjan Elfrink^11^; Michelle Simons^11^; Marjolein Vossers^12^; Joke de Meris^14^; Tamara Vermeulen^14^; Annet Geerlings^19^; Gina van Vemde^22^; Tiny Simons^30^; Gert Messchendorp^28^; Nynke Nicolaij^28^; Hester Bongenaar^32^; Karin Bodde^24^; Sandra Kleijn^34^; Jasmijn Lodico^34^; Hanneke Droste^34^; Maureen Wollaert^5^; Sabrina Verheesen^5^; D. Jeurrissen^5^; Erna Bos^9^; Yvonne Drabbe^15^; Michelle Sandiman^15^; Nicoline Aaldering^11^; Berber Zweedijk^17^; Jocova Vervoort^21^; Eva Ponjee^22^; Sharon Romviel^19^; Karin Kanselaar^19^; Denn Barning^10^; Laurine van der Steen^3^

**Clinical/imaging data acquisition**

Esmee Venema^40^; Vicky Chalos^1,40^; Ralph R. Geuskens^3^; Tim van Straaten^19^; Saliha Ergezen^1^; Roger R.M. Harmsma^1^; Daan Muijres^1^; Anouk de Jong^1^; Olvert A. Berkhemer^1,3,6^; Anna M.M. Boers^3,39^; J. Huguet^3^; P.F.C. Groot^3^; Marieke A. Mens^3^; Katinka R. van Kranendonk^3^; Kilian M. Treurniet^3^; Manon L. Tolhuisen^3,39^; Heitor Alves^3^; Annick J. Weterings^3^; Eleonora L.F. Kirkels^3^; Eva J.H.F. Voogd^11^; Lieve M. Schupp^3^; Sabine L. Collette^28,29^; Adrien E.D. Groot^4^; Natalie E. LeCouffe^4^; Praneeta R. Konduri^39^; Haryadi Prasetya^39^; Nerea Arrarte-Terreros^39^; Lucas A. Ramos^39^; Nikki Boodt^1,2,40^; Anne F.A.V Pirson^5^; Agnetha A.E. Bruggeman^3^; Nadinda A.M. van der Ende ^1,2^, Rabia Deniz^3^, Susanne G.H. Olthuis^5,44^, Floor Pinckaers^6,44^

**List of affiliations**

Department of Neurology^1^, Radiology and Nuclear Medicine^2^, Public Health^40^, Erasmus MC University Medical Center Rotterdam;
Department of Radiology and Nuclear Medicine^3^, Neurology^4^, Biomedical Engineering & Physics^39^, Amsterdam UMC, University of Amsterdam, Amsterdam;
Department of Neurology^5^, Radiology & Nuclear Medicine^6^, Maastricht University Medical Center+; School for Cardiovascular Diseases Maastricht (CARIM)^44^_;_ and MHeNs School for Mental Health and Neuroscience, Maastricht, the Netherlands^45^;
Department of Neurology^7^, Radiology^8^, Sint Antonius Hospital, Nieuwegein;
Department of Neurology^9^, Radiology^10^, Leiden University Medical Center;
Department of Neurology^11^, Radiology and Nuclear Medicine^12^, Rijnstate Hospital, Arnhem;
Department of Radiology^13^, Neurology^14^, Haaglanden MC, the Hague;
Department of Neurology^15^, Radiology^16^, HAGA Hospital, the Hague;
Department of Neurology^17^, Radiology^18^, University Medical Center Utrecht;
Department of Neurology^19^, Neurosurgery^20^, Radiology^27^, Radboud University Medical Center, Nijmegen;
Department of Neurology^21^, Radiology^26^, Elisabeth-TweeSteden ziekenhuis, Tilburg;
Department of Neurology^22^, Radiology^23^, Isala Klinieken, Zwolle;
Department of Neurology^24^, Radiology^25^, Reinier de Graaf Gasthuis, Delft;
Department of Neurology^28^, Radiology^29^, Medical Imaging Center, University Medical Center Groningen;
Department of Neurology^30^, Radiology^31^, Atrium Medical Center, Heerlen;
Department of Neurology^32^, Radiology^33^, Catharina Hospital, Eindhoven;
Department of Neurology^34^, Radiology^35^, Medisch Spectrum Twente, Enschede;
Department of Radiology^36^, Amsterdam UMC, Vrije Universiteit van Amsterdam, Amsterdam;
Department of Radiology^37^, Noordwest Ziekenhuisgroep, Alkmaar;
Department of Radiology^38^, Texas Stroke Institute, Texas, United States of America;
Department of Neurology^42^, Radiology^41^, Albert Schweitzer Hospital, Dordrecht.
Department of Neurology^43^, Amphia Hospital, Breda
